# Adapting and Implementing a Community Program to Improve Retention in Care among Patients with HIV in Southern Haiti: “Group of 6”

**DOI:** 10.1155/2014/137545

**Published:** 2014-12-07

**Authors:** John A. Naslund, Jodie Dionne-Odom, Cléonas Junior Destiné, Kristen M. Jogerst, Redouin Renold Sénécharles, Michelande Jean Louis, Jasmin Desir, Yvette Néptune Ledan, Jude Ronald Beauséjour, Roland Charles, Alice Werbel, Elizabeth A. Talbot, Patrice Joseph, Jean William Pape, Peter F. Wright

**Affiliations:** ^1^The Dartmouth Institute for Health Policy and Clinical Practice, Dartmouth College, Lebanon, NH, USA; ^2^Division of Infectious Diseases, Department of Medicine, University of Alabama at Birmingham, Birmingham, AL, USA; ^3^Centres GHESKIO, Port-au-Prince, Haiti; ^4^Hôpital Immaculée Conception, Les Cayes, Southern Department, Haiti; ^5^Geisel School of Medicine at Dartmouth, Hanover, NH, USA; ^6^United States Centers for Disease Control and Prevention, Les Cayes, Southern Department, Haiti; ^7^Dartmouth-Hitchcock Medical Center, Lebanon, NH, USA; ^8^Division of Infectious Disease and International Health, Department of Medicine, Geisel School of Medicine at Dartmouth, Hanover, NH, USA; ^9^Center for Global Health, Division of Infectious Diseases, Department of Medicine, Weill Cornell Medical College, New York, NY, USA

## Abstract

*Objective*. In Mozambique, a patient-led Community ART Group model developed by Médecins Sans Frontières improved retention in care and adherence to antiretroviral therapy (ART) among persons with HIV. We describe the adaptation and implementation of this model within the HIV clinic located in the largest public hospital in Haiti's Southern Department. *Methods*. Our adapted model was named Group of 6. Hospital staff enabled stable patients with HIV receiving ART to form community groups with 4–6 members to facilitate monthly ART distribution, track progress and adherence, and provide support. Implementation outcomes included recruitment success, participant retention, group completion of monthly monitoring forms, and satisfaction surveys. *Results*. Over one year, 80 patients from nine communities enrolled into 15 groups. Six participants left to receive HIV care elsewhere, two moved away, and one died of a non-HIV condition. Group members successfully completed monthly ART distribution and returned 85.6% of the monthly monitoring forms. Members reported that Group of 6 made their HIV management easier and hospital staff reported that it reduced their workload. *Conclusions*. We report successful adaptation and implementation of a validated community HIV-care model in Southern Haiti. Group of 6 can reduce barriers to ART adherence, and will be integrated as a routine care option.

## 1. Introduction

In Haiti, the availability of antiretroviral therapy (ART) has increased, yet in 2012 fewer than 60% of patients with HIV received this life sustaining treatment [1]. The clinical effectiveness of ART depends on retention in care and long-term adherence [2]. However, in Haiti, fewer than half of patients newly diagnosed with HIV who are eligible for ART initiate treatment within 2 years [3], and retention rates one year after initiating ART are below 80% [4]. These low retention rates are likely due to stigma, lack of understanding about HIV or ART, work and family commitments, long clinic wait times, and poverty, which makes high transportation costs to distant ART facilities a significant burden [5]. Further, inadequate staffing and few resources at health care facilities dedicated to improving retention in care contribute to these low rates.

Similar barriers to retention in care are found in resource-limited settings throughout sub-Saharan Africa [6–8]. A systematic review of 26 studies identified several interventions for promoting ART adherence [2]. These included patient education, individual or group counseling, direct observation of therapy, additional treatment support and case management, mobile-phone text message reminders, and material support such as transportation reimbursement or food supplements [2]. Despite the initial success of many of these interventions, concerns have been raised about whether the effects are long lasting, fiscally sustainable, or generalizable across diverse settings. Many interventions require hiring additional staff in clinics or communities and purchasing drugs, equipment, and materials that are not feasible in severely resource-constrained settings.

More recently, emphasis has been placed on community participation and involving patients to support ART delivery [9–12]. Shifting medical tasks to patients incorporates care processes into their daily lives, which is essential to promoting lifelong adherence [13]. Additionally, community-based models that leverage the expertise of persons living with HIV to promote ART adherence require fewer resources to implement and sustain, can achieve widespread reach, and can reduce workload for providers and health systems [9, 14].

In rural Mozambique, the Community ART Group (CAG) model has emerged as a highly promising example of patient-led ART provision, initially described by Decroo et al. [15]. The CAG model was implemented and evaluated by Médecins Sans Frontières (MSF) and the district Ministry of Health and has improved retention in care [16]. In this model, patients with HIV who are stable on ART for at least 6 months and have a CD4 count over 200 cells/mL form peer groups of up to 6 members. On a monthly rotational basis, one member from each group visits the clinic to collect ART for the other group members and to attend a clinic visit with a physician. This process rotates such that each group member attends a required follow-up visit every six months. Back in the community, the visiting group member then distributes the ART to the other group members. The groups also participate in activities for adherence monitoring and for outcome reporting, as well as for social support.

Our present work is devoted to determining whether the CAG model can be adapted and implemented in one setting in Haiti and whether it can improve retention in care. We provide an overview of our process of adapting the CAG model and the key considerations from stakeholders when creating the revised version of the program which we named “Group of 6.” Then, we outline the implementation of the Group of 6 program. Lastly, we present our one-year implementation outcomes, lessons learned, and next steps.

## 2. Program Adaptation

### 2.1. Project Setting

Haiti's Southern Department has a population over 700,000, of which about 80% live in rural areas [18]. Adult HIV prevalence in the Southern Department is estimated at 2.2% [19]. There are about 11 health facilities in the Department where ART can be obtained [20], the largest of which is the President's Emergency Fund for AIDS Relief (PEPFAR) Clinic located at Hôpital Immaculée Conception (HIC), the central public hospital operated by the Haitian Ministry of Health and located in Les Cayes, the Department's largest city. The current study was conducted through HIC, which has about 3,000 patients with HIV enrolled in care, of which 62% are taking ART supported by PEPFAR funding [20]. In the two-year period prior to starting this project from 2010 to 2012, the average adherence among enrolled patients at HIC was 70.8% [20].

As part of national efforts to improve retention in care, the Haitian Group for the Study of Kaposi's Sarcoma and Opportunistic Infections (GHESKIO) in collaboration with the United States Centers for Disease Control and Prevention (CDC) has been monitoring and supporting HIV care at HIC since 2003. Previously established efforts to improve retention in care among patients with HIV in the Southern Department have included the use of community health workers, called “agents de terrain,” who are employed by the Ministry of Health to support HIV treatment efforts such as identifying and encouraging at-risk individuals to come to HIC for testing and to enroll in care, as well as following up with patients from distant communities who are lost to follow-up. Additionally, there have been significant efforts to improve data collection and monitoring of patient outcomes through the development and implementation of the iSanté database supported by PEPFAR and the CDC. To our knowledge, no community-based interventions targeting retention in care similar to the CAG model have been previously implemented at HIC.

### 2.2. Adapting the Mozambique CAG Model

We initiated this project as part of a CDC funded grant aimed at supporting the PEPFAR goals related to HIV treatment and prevention at HIC [21]. The project is overseen by GHESKIO Centers in Port-au-Prince, Haiti [22]. In early 2012, following approval of the hospital director, we convened a stakeholder meeting to present the CAG model to the health care providers at HIC as a program for improving retention in care among stable patients on ART. Stakeholders reported that the CAG model was needed and they believed that it could be successfully implemented and sustained over time given the few resource requirements. The health care providers estimated that about 47% of patients enrolled in care at HIC and 76% of those who were active in care would be eligible.

We then convened a second larger stakeholder meeting to obtain feedback regarding the steps necessary for successful adaptation and implementation of the CAG model. This meeting included the HIV clinic social worker, the pharmacist, 19 community health workers, the clinic supervising nurse, one of the clinic physicians, and 5 community nurses. There were also five patients receiving ART at HIC who were present at this meeting, and they offered valuable perspectives from the patients' point of view. Two members of the research team, John A. Naslund and Cléonas Junior Destiné (who is also the project physician and a member of the HIV clinic care team at HIC), facilitated the meeting.

The first major concern that emerged during the stakeholder meeting was the low literacy rate in rural Haiti (according to a 2013 UNICEF report, the total adult literacy rate was 48.7% [23]). The health care providers and community health workers advocated for simplified monitoring forms and novel approaches to ensure that ART would be correctly distributed among illiterate group members. Therefore we created a simplified monthly monitoring form in Haitian Creole. In addition to having group members record a monthly pill count, we added questions to monitor for symptoms and to assist the project physician with tracking group members' progress. Based on stakeholder input, we made it a requirement that at least one member enrolled in each group be literate in order to assist with pill counts and completion of the monthly monitoring forms. We also assigned colors (red, blue, yellow, purple, green, or brown) to each group member at enrollment. The pharmacist used the colors to individually mark the ART containers for each participant when bundling the group's ART together for distribution in the community.

Secondly, concerns related to stigma were extensively discussed. The complexity of stigma in the region was highlighted when community health workers explained that there are cases in which both partners in a married couple are HIV positive, yet neither is aware of the other's status. They also mentioned circumstances where, in an attempt to remain anonymous, patients choose to travel very long distances (up to 90 km each way) to obtain ART at HIC rather than to visit smaller nearby treatment facilities or dispensaries. It was clear that requiring groups to self-form, as in the CAG model, would not be feasible due to stigma. Therefore, a major adaptation to the CAG model was elected by stakeholders, in which the social worker and community health workers would identify interested and eligible patients who live in close proximity to one another and then offer them participation in a group. The need to disclose HIV status to other group members was clearly presented to potential participants. Through membership in a group, participants would discover that others in their community shared their diagnosis and would have a forum for confidential discussion about their experience with HIV. Among CAG participants in Mozambique this has led to reductions in HIV-related stigma [17, 24]. To avoid inadvertent disclosure of HIV status to nongroup members, stakeholders also advocated that each participant's ART is packaged into opaque plastic bags to conceal the contents for distribution in the community and that each group is provided with a simple black backpack to conceal and securely transport the ART.

### 2.3. CAG Model to Group of 6

We named our version of the CAG model “Group of 6,” known as* Gwoup 6* in Haitian Creole. The agreed design is that, on a monthly basis, the group members arrange to meet in their community to complete the monthly monitoring form. This form records pill count, the number of pills missed during the last 30 days, symptom tracking, and plans to stay in the group. The group completes this task together, and each member must acknowledge that they were present by marking an “X” on the form (an “X” was used instead of a signature to hide the identity of the group members if the form was lost). The monthly monitoring forms serve to ensure accountability among group members, to allow clinicians at HIC to monitor group progress, and to confirm that ART was distributed correctly among members each month.

Then, on a rotational basis, one member from each group visits HIC to collect ART for all of the other group members. During this monthly visit to the hospital clinic, the member sees a physician for routine follow-up. Thus each group member visits HIC at least once every 6 months for HIV care and assessment of their HIV status. During visits to HIC, the group member also reviews group progress, which was documented in the group monthly monitoring form. The physician or social worker then specifically guides the member to discuss whether there are any concerns with adherence or challenges with the group process. The group member also meets with the pharmacist to obtain his/her ART as well as the ART packaged in color-coded bags for all of his/her other group members. Once back in the community, the group member distributes the ART to the other members of his/her group.

Participation in the Group of 6 program was voluntary and was not intended to replace care for acute medical illness. Patients who elected to participate in the Group of 6 program were counseled and reminded that they should visit HIC when unwell or for other health concerns.

## 3. Program Implementation

**Figure 1:**
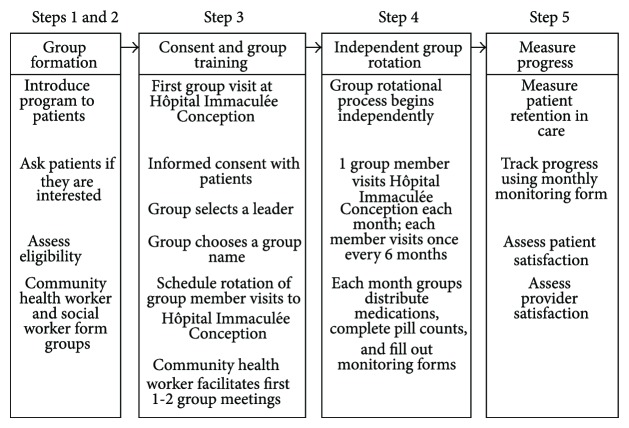
Steps in the Group of 6 program.

Eligible participants were adults (age 18 and older), clinically stable on ART for a minimum of 6 months, without any active opportunistic infections, and with a CD4 count of at least 200 cells/mL. Implementation of Group of 6 was conducted in five steps as outlined in Figure 1. Step 1: the Group of 6 program concept was introduced to patients during routine follow-up visits at HIC with a physician or with the clinic social worker or during routine community health worker visits in their communities. Interested patients were assessed for eligibility by review of their clinical records in the PEPFAR iSanté electronic database. Step 2: the social worker, community health workers, or project physician described the details of the Group of 6 program to interested and eligible patients, including the need to disclose one's HIV status to other group members. Based on their knowledge of the geography of the Southern Department, the social worker and community health workers organized interested and eligible patients into potential groups of 4–6 members based on proximity to one another. Then, these suggested groups were encouraged to come to HIC at their earliest convenience to formally enroll in the Group of 6 program. The social worker and community health workers made considerable efforts to recruit and enroll groups of participants from distant communities, given the challenges in retaining these individuals in care.

Step 3: newly formed groups met at HIC with the project physician and social worker to complete informed consent. During this meeting, each group selected a group leader who would oversee completion of the monthly monitoring forms and pill counts in the community. Each group was then encouraged to choose a group name to personalize the project and promote ownership among group members. This was a very popular part of the initial group meeting and helped to form group cohesiveness. After reviewing the instructions for the group rotational process, group members practiced conducting pill counts and filling out the monthly monitoring form with the physician or social worker observing. Next, each group member chose a color that would be used to mark their ART for distribution each month, and with the help of the pharmacist, the group prepared a schedule for rotational visits for the coming months. The community health workers were instructed to supervise the first 1-2 group meetings in the community to ensure that ART was distributed correctly to all group members.

Step 4 of implementation consisted of full autonomy of the Group of 6, where rotations proceeded independently as intended without additional support from the community health workers. Step 5: the progress and implementation of the Group of 6 program were evaluated. The primary measures of implementation were rate of group formation, retention in care, success of the group process as reflected by the proportion of completed monthly monitoring forms for each group, and general satisfaction with the program as reported by participants and providers. To determine retention in care, we recorded reasons why participating patients were lost to follow-up. We tracked completion of the monthly monitoring forms for each group and considered the successful completion of these forms as an indicator of effective program adherence and understanding among participants. Interest among patients not yet enrolled was determined through discussions with the social worker and community health workers, and rate of recruitment was used as an indicator of successful program implementation. Lastly, we used a two-question survey to assess patient satisfaction with the Group of 6 program. Surveys were administered during group members' monthly visits to HIC and were kept brief due to time constraints in the clinical setting. Similar surveys were administered every 6 months to hospital staff at HIC, including health care providers and community health workers, involved in supervising and delivering the program.

### 3.1. Consent Procedures and Ethics

All study procedures for enrollment, implementation, and evaluation of Group of 6 were approved by Committees for the Protection of Human Subjects at GHESKIO Centers in Port-au-Prince, Haiti, and at Dartmouth College, Hanover, NH, USA. The consent forms were translated into Haitian Creole and were read to patients within their groups by the project physician or social worker at HIC. Interested and eligible patients then provided informed, individual consent. An additional witness, usually a community health worker or the social worker, was present to assure comprehension when consenting patients were unable to read or write.

## 4. Results

Adaptation of the CAG model to the Group of 6 program was successful. The unique program name was popular among the health care providers and community health workers at HIC because it demonstrated local ownership of the program. Choosing individual group names was also observed to be a very popular part of the initial group meeting and helped to form group cohesiveness among participants.

**Figure 2:**
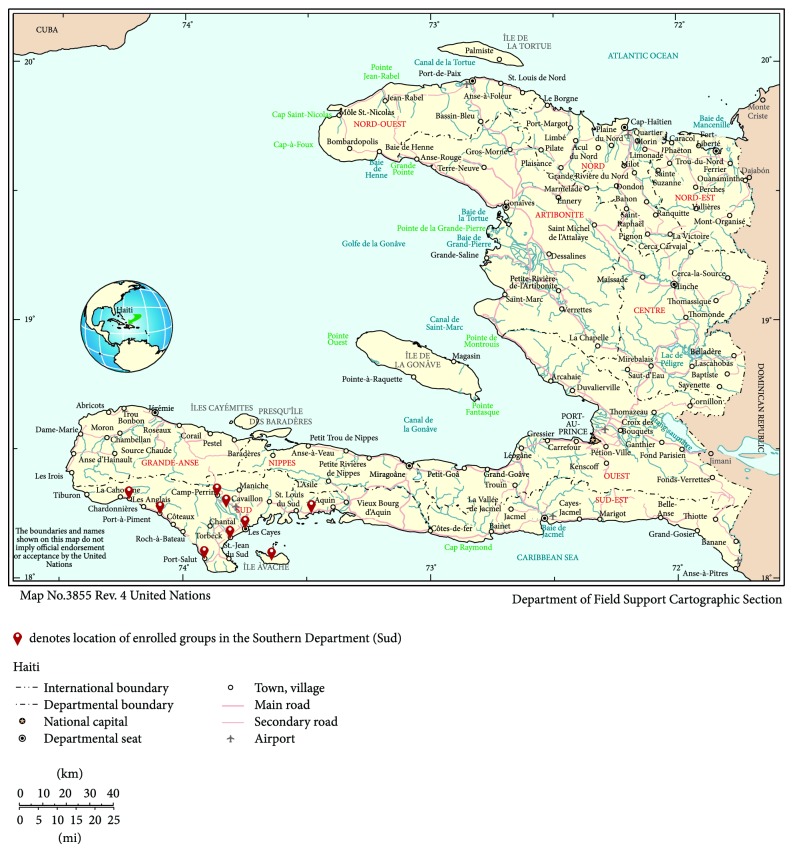
Geographic distribution of groups enrolled in the Group of 6 program throughout Haiti's Southern Department. Source: this map was adapted from United Nations Map No. 3855 Rev. 4: United Nations Department of Field Support Cartographic Section; 2008 [cited 2014 May 31]. Available from http://www.un.org/depts/Cartographic/map/profile/haiti.pdf [17].

Between January 2013 and March 2014, 80 patients were enrolled into 15 groups. The baseline characteristics of participants are listed in Table 1. The groups were comprised of 4 to 6 members and were from 9 different communities up to 78 km from HIC. The geographic distribution of the groups is shown in Figure 2 [25]. The clinic team was able to recruit seven groups from distant communities between 24 km and 78 km from HIC, which was considered a success given that there are numerous challenges in reaching these individuals and retaining them in care. Even short distances from HIC can be difficult to travel because of the poor condition of the roads, frequent flooding, mountainous terrain, and limited and costly transportation options.

Among the 80 participants, retention was 88.4%, with participants remaining engaged for 658 out of a possible 744 months enrolled. Two participants left their groups because they moved out of the Southern Department, and 1 participant died due to a non-HIV related chronic condition. One group with 6 participants left the program because HIV care was made available within their community on Île-à-Vache, a several hour boat ride from HIC. The remaining 71 participants (88.8% of our sample) continue to participate in the program.

Through follow-up discussions with the community health workers and social worker, as well as the monthly follow-up visits with the visiting member for each group, we confirmed that group members correctly distributed the ART within their respective communities. Two groups experienced some initial confusion regarding the monthly rotational process and required assistance from the community health workers before resuming independent participation. These concerns were addressed through follow-up meetings with group members and additional visits by the community health workers within the participants' community. A total of 95 out of a possible 111 monthly monitoring forms (85.6%) were completed and returned. Eleven of the missing forms were from the 2 groups who experienced confusion early in the process.

The social worker and community health workers have continued to inform new patients about the Group of 6 program and have made ongoing efforts to reach patients in distant communities. There appears to be considerable interest among patients with HIV seen at HIC, although a number of challenges have delayed recruitment of new groups. For example, recruitment of new groups was hindered by heavy rains, which made it more difficult to travel to HIC from remote communities. Additionally, transportation costs pose major barriers to recruitment because participants report that they cannot afford the cost of traveling to HIC for the initial recruitment visit. We provided reimbursement for the initial recruitment visit to HIC, but paying the costs up front has been a barrier for many patients.

Satisfaction survey data from participants and hospital staff are shown in Table 2. Forty-eight participants completed the satisfaction survey. Patients reported satisfaction with the program, indicating that they agree (35%; *N* = 17) or strongly agree (65%; *N* = 31) that the Group of 6 program has helped them take care of themselves. Patients also stated that they agree (42%; *N* = 20) or strongly agree (58%; *N* = 28) that the program has helped them feel more confident managing their HIV. Five providers, including the social worker, the pharmacist, and 3 community health workers, also completed the satisfaction survey. Hospital staff expressed strong satisfaction with the Group of 6 program, with all five respondents reporting that it has decreased their clinic workload. Among hospital staff, one agreed and three strongly agreed that the program has been helpful to their patients, while one was neutral cautioning that it may be too early to draw conclusions regarding clinical outcomes.

## 5. Discussion

We have successfully modified and implemented the patient-led Community ART Group (CAG) model from Mozambique for a largely rural, low-resource setting in Southern Haiti. Our 1-year implementation outcomes are promising, as reflected by our enrollment of 15 groups and our preliminary finding of high retention in care among participants. Those few who left the program did not leave because of dissatisfaction with Group of 6.

A key factor in the successful implementation of Group of 6 was ensuring that stakeholders including health care providers, community health workers, and patients own the program. This was achieved through the involvement of stakeholders at an early stage in project planning, beginning with making decisions and providing suggestions surrounding the program adaptation and delivery and further by naming the adapted model “Group of 6.” Among newly enrolled groups of patients, the opportunity to select a group name served as a way to establish a personal connection with the group and form a group identity. All 15 groups chose creative names, reflecting the pride they take in their association, for example,* Chache La Vi* (Pursuit of Life),* Tet Ansanm* (All Together),* Fanm Vanyan* (Courageous Women),* Espwa* (Hope), or* Union* (Union). The importance of fostering ownership of community-based initiatives has been discussed as vital for successful program delivery and sustainability [9].

Institutional characteristics were also an important factor that may have contributed to the successful implementation of the Group of 6 program. First, support from the hospital director helped to create a favorable hospital climate [26]. Also, the enthusiasm toward implementing Group of 6 expressed by several of the health care providers and community health workers was critical for advancing program implementation and sustainability. These individuals, referred to as “implementation leaders” or “local champions” [27], played an essential role in implementing the program. For example, several community health workers who themselves were taking ART at HIC were early adopters of the program and were instrumental in mobilizing patients and enrolling the first groups of participants. These individuals helped by demonstrating the benefits of Group of 6 to others who were more hesitant or less certain. Others soon followed as the program became more established and trusted. This observation is consistent with other research that shows that when those who promote the implementation of an intervention are similar to or can easily relate to the target population, then implementation will be more successful [28]. This may have been the case with these local champion community health workers, because they were able to relate to study participants.

The monthly monitoring forms were an important measure of adherence to the Group of 6 program and served as an indirect measure of program fidelity. Measuring the fidelity of newly implemented interventions can be difficult due to time or resource constraints [29]. These forms served as a feasible measure because they were collected as part of regular program delivery, without additional data collection costs or requirements. Completed forms helped to confirm that group members were meeting in the community independently, were correctly distributing medications each month, and were accountable to each other. Our return rate of 85.6% of the monitoring forms suggests excellent adherence to the program. When forms were incorrectly completed or were not returned at all, the project physician and social worker were alerted about groups that were struggling, which prompted additional support and instruction as necessary.

We acknowledge that there are several limitations with the results reported here. First, while our data show good retention in care among participating patients, it is not possible to determine whether the Group of 6 program contributed to these high retention rates because we did not have baseline retention in care data for these participants and we did not have a control group to rule out temporal trends or selection bias. We believe that the Group of 6 program can improve retention in care among participating patients because research has shown that when resources and efforts are dedicated to addressing barriers to care such as transportation costs, improvement in retention can be achieved [30], though additional follow-up is necessary. Second, it is possible that because our participants were stable on ART and joined the program voluntarily, they may have been more likely to remain in care than other patients with HIV seen at HIC regardless of the Group of 6 program. Therefore, our preliminary findings are not generalizable to new patients or to a wider group of patients seen at the HIV clinic at HIC or elsewhere in Haiti. However, given that the Group of 6 program requires few additional resources and offers the potential to reduce workload for health care providers, this may represent a valuable approach for retaining stable patients in care, thereby allowing providers to focus on more at-risk patients.

Third, this study was preliminary in nature and it is therefore too early to confirm whether the Group of 6 program offers clinical benefit in terms of improved outcomes related to morbidity and mortality. We are currently conducting a quasiexperimental evaluation of the effectiveness of the Group of 6 program in comparison to a matched control group consisting of patients meeting the same eligibility criteria but who are not enrolled in the program. Fourth, we cannot answer what uptake there would be if this program were offered to every patient. Because our recruitment strategy involved widely communicating with patients through the clinic at HIC and in the patients' communities during community health worker visits, we cannot determine how many patients were approached to participate in the Group of 6 program, nor can we determine the proportion who refused participation.

Additionally, our measure of satisfaction was subject to desirability bias given that patients completed it during routine visits to HIC with clinicians present who were involved in the current project. We will also conduct in-depth semistructured follow-up interviews with group participants led by an objective interviewer to better understand the impact of group participation on social support, perceived stigma, overcoming transportation, or other barriers to care and to elicit additional perspectives from participating patients. Lastly, it is not possible to fully document that the Group of 6 program helped reduce staff workload in spite of the responses to the satisfaction surveys completed by health care providers and community health workers at HIC. As a next step, we will use the PEPFAR iSanté electronic patient database to record the number of clinic visits among group members compared to patients in the matched control group.

While we hope that Group of 6 will eventually help reduce stigma in the region, it is clear that stigma was a considerable obstacle to participant recruitment. According to the clinic social worker and community health workers, many patients were reluctant to form or participate in groups because of concerns related to disclosing their HIV status. In addition, the cost of transportation in the Southern Department remained a significant barrier. Even though Group of 6 in sum reduces participants' total travel costs and we provided reimbursement for the initial recruitment visit at HIC, we observed that the cost of transportation remained a deterrent to participation. Lastly, inclement weather emerged as a substantial barrier to participant recruitment. Heavy rains made it very difficult for members of a new group from a distant community to travel to HIC in order to enroll in the project. We are exploring strategies to facilitate recruitment in such circumstances, such as staggering recruitment where groups can enroll with 3 or 4 members before adding other interested and eligible participants.

Overall, the Group of 6 program remained consistent with the primary objectives of the CAG model to improve retention in ART [15, 16, 31]. Eligibility criteria were largely unchanged, and the basic principles of rotational visits to a central health facility to obtain ART for distribution to the entire group were the same. Our program adaptations align with many views in implementation research that intervention adaptations are necessary for successful implementation as long as the “core elements” of the program remain intact [29].

## 6. Conclusion

Implementing a new model of care is a highly complex process, especially in a rural, resource-limited setting. This report contributes our experience and offers valuable insight for future efforts to adapt and implement community programs in such settings. To date, implementation research in resource-limited settings has received inadequate attention [32]. Our imperative to implement a well-validated model of care from another resource-limited setting stemmed from the immediate need for strategies to overcome barriers to ART adherence [33]. In our context of HIV care delivery in Haiti's Southern Department, these barriers include poverty, stigma, work or family commitments, and high transportation costs [30].

We demonstrated that a model of HIV care that was pioneered in sub-Saharan Africa is relevant and applicable in Southern Haiti. Our findings suggest that novel ART delivery models, such as the CAG model, can be readily adapted to the local context and available resources [17]. Following our initial success in implementing the Group of 6 program at HIC, we anticipate that it can be integrated into usual care. Next steps will include providing ongoing and sustainable support to existing groups and scaling up the program to reach more patients. Our research team is currently working in collaboration with GHESKIO Centers and other HIV clinics in Southern Haiti as well as in other departments to roll out this program to other sites. Our findings illustrate that models that leverage patients as active participants and that require few additional resources are adaptable, viable for implementation, and potentially sustainable.

## Figures and Tables

**Table 1:** Baseline characteristics of Group of 6 participants.

Category	Value
Total patients enrolled	80
Number of groups	15
Number of geographic areas with groups	9
Average number of patients per group	5.3
Female, number (%)	51 (63.8%)
Age at enrollment (years), median (IQR)	44 (36–51)
CD4 count at enrollment (cells/mL), median (IQR)	509 (382–670)
Months on ART prior to enrollment, median (IQR)	45.2 (22.4–65.6)

IQR: interquartile range.

**Table 2:** Responses to participant and hospital staff satisfaction questionnaires.

Satisfaction survey questions^a^	Mean	SD^b^
Participants (*N* = 48)		
(1) Participating in the Group of 6 program has made it easier for me to take care of myself	1.35	0.48
(2) I feel more confident managing my own health condition when compared to before participating in the Group of 6 program	1.42	0.50
Hospital staff (*N* = 5)		
(1) By having my HIV positive patients participate in the Group of 6 program, it has helped reduce my workload in the clinic or in the community	1	0
(2) Among my patients participating in the Group of 6 program, I have observed an improvement in their ability to manage their HIV	1.60	0.89

^a^Possible scores were 1 (strongly agree), 2 (agree), 3 (neither agree nor disagree), 4 (disagree), and 5 (strongly disagree).

^b^Standard deviation.
